# Semantic gradients in picture-word interference tasks: is the size of interference effects affected by the degree of semantic overlap?

**DOI:** 10.3389/fpsyg.2014.00872

**Published:** 2014-08-12

**Authors:** James Hutson, Markus F. Damian

**Affiliations:** School of Experimental Psychology, University of BristolBristol, UK

**Keywords:** spoken production, picture-word interference, lexical access, object naming, competition

## Abstract

We report two experiments attempting to identify the role of semantic relatedness in picture-word interference studies. Previously published data sets have rendered results which directly contradict each other, with one study suggesting that the stronger the relation between picture and distractor, the more semantic interference is obtained, and another study suggesting the opposite pattern. We replicated the two key experiments with only minor procedural modifications, and found semantic interference effects in both. Critically, these were largely independent of the strength of semantic overlap. Additionally, we attempted to predict individual interference effects per target picture, via various measures of semantic overlap, which also failed to account for the effects. From our results it appears that semantic interference effects in picture-word tasks are similarly present for weakly and strongly overlapping combinations. Implications are discussed in the light of the recent debate on the role of competition in lexical selection.

## Introduction

Models of language production which incorporate competitive lexical selection (Roelofs, 1992; Caramazza, 1997; Levelt et al., 1999; La Heij et al., 2006) have recently been challenged by claims that selection occurs without competition (e.g., Mahon et al., 2007). A major source of evidence supporting competitive processing in language production arises from an empirical effect found in picture-word interference (henceforth PWI) experiments. The *semantic interference effect* is characterized by the slower naming of a picture (e.g., *bear*, “target”) when a superimposed written (or simultaneously spoken) word (“distractor”) is related in meaning (e.g., *whale*) in comparison to when it is unrelated (e.g., *house*). The increased difficulty in selecting the picture name in the presence of a semantically related item was long thought to imply competitive lexical selection (e.g., Roelofs, 1992, 1993, 2001, 2003; Humphreys et al., 1995; Starreveld and La Heij, 1995, 1996; Costa et al., 1999; Damian and Martin, 1999; Levelt et al., 1999; Vitkovitch and Tyrrell, 1999; Caramazza and Costa, 2000; Bloem and La Heij, 2003; Damian and Bowers, 2003; Vigliocco et al., 2004; Belke et al., 2005; Hantsch et al., 2005, 2009). However, this view has recently been challenged (e.g., Finkbeiner and Caramazza, 2006; Mahon et al., 2007; Janssen et al., 2008). The work reported in this article investigates the issue via the question whether the degree of semantic overlap between target and distractors is relevant in PWI tasks.

Lexical selection serves the purpose of isolating the single most appropriate item from a cohort of related items. Spoken word production is initiated at the level of conceptual preparation. A cohort of items, often referred to as nodes, is said to become active because activation spreads between related concepts and their components (e.g., Levelt, 1999). Thus, when presented with a picture of a *bear*, for example, other related items such as *wolf*, *deer*, and *rabbit* (among others), will form the related cohort, with the activation level of each item being determined by the strength of semantic relationship with the target. Activated cohort items at the conceptual level subsequently spread activation to corresponding nodes at the lexical level (Collins and Quillian, 1969; Collins and Loftus, 1975; Dell, 1986; Roelofs, 1992; Caramazza, 1997; Levelt et al., 1999, but see Bloem and La Heij, 2003). Selection of a single item from the activated cohort must then take place at the lexical level prior to further processing.

Semantic interference effects in PWI tasks have long been attributed to the co-activation of the distractor word's representation at the lexical level, delaying the retrieval of the target's name (e.g., La Heij, 1988; Glaser and Glaser, 1989; Schriefers et al., 1990; Vitkovitch and Humphreys, 1991; Roelofs, 1992; Levelt et al., 1999). Indeed, semantic interference of this type initially motivated the inclusion of competitive selection principles in models of spoken production. For instance, in one of the most prominent models of language production, WEAVER++ (Roelofs, 1992; Levelt et al., 1999), the speed with which selection of a target item takes place is determined by its activation level in relation to the summed activation level of all other active lemmas. Hence, targets and non-targets compete: the greater the target's activation level compared to other items, the faster it will be selected, and co-activated non-target items slow response selection. Semantic interference in PWI is accounted for via an exchange of activation between target and distractor at the semantic level (see Roelofs, 1992, for detailed computational simulations).

Recently, however, this interpretation has been challenged, based on results from a variety of methods (see Mulatti and Coltheart, 2012; Spalek et al., 2012, for recent reviews). Instead, an alternative account of PWI effects has been introduced, the “response exclusion hypothesis” (REH, see Mahon et al., 2007, for the most detailed outline of this position). According to this view, it is not lexical competition between target and distractor which causes semantic interference in PWI, but instead, REH advocates a post-lexical, articulatory locus of inhibition. Distractors come to occupy a prearticulatory buffer from which they must be removed prior to the commencement of target naming. In REH, task demands set the rules which govern the ease with which distractors can be removed from the buffer. The semantic component governing task demands is relatively crude and operates on the fulfillment of broad semantic constraints such as category membership, but it is insensitive to more fine-grained semantic properties. Thus, distractor words that share category membership with target pictures are more difficult to remove from the buffer than those which do not. This increased difficulty, according to REH, results in the categorical interference witnessed in numerous picture-word experiments.

The general idea that the distractor needs to be removed from a response buffer before target naming can proceed was initially motivated by the observation that when the frequency of distractor words is manipulated, low frequency distractors generate more interference than high frequency ones (Miozzo and Caramazza, 2003). This finding seems difficult to reconcile with the notion of lexical selection by competition, as the latter view would either predict the opposite pattern, or a null finding of distractor frequency. A further central observation in recent research is that semantic interference appears restricted to categorically related pictures and distractors; other forms of overlap (e.g., part-whole, associations, etc.) tend to generate facilitation (Bloem and La Heij, 2003; Costa et al., 2005). On a competitive account, it is not immediately clear why type of overlap should be of such major relevance. If interference arises as a result of overlap at the semantic level, then all sorts of semantic relations should result in similar effects. As outlined above, the REH advocates a “response buffer” locus of the effect which is sensitive to only very broad semantic criteria. Hence, categorically related distractors have sufficient “task relevance” to delay removal of the distractor word from the response buffer, whereas non-categorically related distractors (e.g., target: *mouse*; distractor: *cheese*) are not interpreted as task-relevant and so create no interference compared to unrelated items. In order to account for the facilitation (rather than interference) typically caused by non-categorical relationships, REH advocates that this reflects priming at the conceptual level. Hence, the net behavioral effect in PWI tasks is taken to result from a combination of semantic priming on the one hand, and response-buffer-based interference on the other hand which is restricted to categorically related pairs (see Blackford et al., 2012, for recent EEG-based evidence that semantic effects in spoken word productions could arise from multiple sources). By abandoning a competitive principle active in lexical selection, REH constitutes a major break with conventional thinking about this issue. However, it must be noted that there are alternative scenarios that maintain the notion of competitive lexical selection while still accounting for the observation that semantic interference in PWI is restricted to category members. Abdel Rahman and Melinger (2009) have suggested that the flow of activation in the conceptual stratum is very different when items are related categorically and noncategorically. The former is characterized by the activation of a large cohort of items with similarly high levels of activation; the latter, by a comparatively small number of items with a greater range of activation. Thus, interference is restricted to items sharing category membership while other forms of meaning overlap result in conceptual priming.

In the work reported below, we focused on the effects of semantically related distractors, and we asked whether the *degree of semantic overlap between target and distractor* has an influence on the size of the resulting interference effect. As will be shown, competitive and non-competitive theories of lexical selection in word production make opposing predictions in this regard, and indeed, two previous relevant data sets directly contradict each other. We will begin with a brief review of the relevant findings.

Very few studies have directly manipulated the semantic distance between distractor and target using the picture-word paradigm. The first investigation of semantic distance in PWI tasks that we are aware of provided evidence for a gradient such that greater target-distractor semantic relatedness resulted in stronger response inhibition. Vigliocco et al. (2004) introduced the “featural and unitary semantic space” (FUSS) hypothesis, a theory which emphasizes the role of featural representations as essential components of conceptual structure. According to this theory, featural representations are bound into lexico-semantic representations, the organization of which is determined by shared and correlated features between concepts. As such, these computational principles can be used to index the relatedness between individual concepts. The authors gathered featural data for a large number of concepts by asking participants to generate a sufficient number of features for each word, and trained a self-organizing computational model on these features, resulting in a semantic map. Vigliocco et al. then used the semantic distance between words/concepts to predict behavioral effects in various experimental paradigms. In their third experiment, they used relatedness scores derived from FUSS to select targets and distractors for a PWI experiment. Of the four conditions, in one (the “far” condition) pictures and distractors were essentially semantically unrelated, while in the other three they varied in the degree of semantic overlap from “medium” to “very close.” Results showed a graded semantic interference effect, such that interference decreased with semantic distance. Furthermore, category membership as such appeared less relevant, as the results did not significantly change when analysis was restricted to only category coordinates.

The finding of a semantic gradient in PWI tasks, with larger interference caused by strongly than by weakly related distractors, is generally compatible with models of competitive lexical selection: highly related target-distractor pairs should engage in a large degree of activation exchange via the conceptual level, resulting in strong competition between distractor and target at the lexical level; weakly related pairs should result in relatively less competition. More recently, however, results were reported which suggest the opposite pattern. Mahon et al. (2007) carried out a series of experiments investigating the effect of manipulating the semantic distance between target pictures and distractor words on the speed of naming times. The first relevant study (Experiment 4) attempted to control for semantic distance while manipulating category membership. Semantic distance values were derived from the semantic similarity norms of Cree and McRae (2003). Relatedness values were established through the number of shared features between items. Participants generated lists of features, in a fashion very similar to the method used by Vigliocco et al. (2004). For example, in response to the word *knife*, participants might have generated the features: “is sharp,” “has a handle,” “used for cutting,” and “found in kitchens.” Items from within a particular category will normally share more features than they do with those from other categories. However, within-category items sometimes share very few features, making it possible to match within- and between-category items on semantic relatedness. Thus, stimuli were constructed in which within-category targets and distractors had the same semantic overlap as targets and distractors from separate categories. It was found that categorically related items caused more interference than non-categorically related items, suggesting that category membership exerted an effect over and above that of semantic relatedness, at odds with the findings of Vigliocco et al. outlined above.

In a subsequent experiment which is particularly critical for the current work, Mahon et al. (2007 Experiments 5 and 5b) directly manipulated within-category semantic distance. Rather than semantic relatedness values derived from the extent of feature overlap, ratings of semantic relatedness from human participants were gathered for each pair of items used in the experiment. Each target picture was paired with a closely related distractor word and a distantly related distractor, or with two unrelated distractor words. Surprisingly, results indicated that distantly related target-distractor pairs (e.g., *horse-whale*) interfered *more* than closely related pairs (*horse-zebra*). This finding was replicated in a further study (Experiment 5b) with the same materials but a separate group of participants. Further support for the direction of the effect was provided in Experiment 6 of the series, where naming times of targets in the context of close and far distractors were compared directly rather than with targets with unrelated distractors superimposed. The effect was reliable by participants (*p* ≤ 0.05) but only marginally by items (*p* = 0.11). In the final two experiments carried out by Mahon et al. (Experiments 7 and 7b) stimuli were selected such that close and far conditions had a large difference in relatedness according to the norms of Cree and McRae (2003). The nature of the semantic relationships generated from the norms was confirmed through ratings obtained from a group of native English speakers. The interval between onset of picture and distractor (stimulus-onset asynchrony, or SOA) was varied; by varying the “entry time” of the distractor relative to that of the target, the distractor taps into successive stages of target presentation, hence yielding information about temporal patterns. Three separate SOAs were examined: −160, 0, and +160 ms. A reliable effect in which distantly related distractors interfered more than closely related distractors was found in both of the staggered presentation conditions but not in the simultaneous presentation condition. A replication of the experiment with only the simultaneous condition again found no effect of semantic distance on naming times for synchronous presentation.

Although the final two experiments in the series carried out by Mahon et al. (2007) provide somewhat ambivalent results, overall the findings suggest increased interference from distractors that are more distantly related targets, compared to those more closely related. The findings of Experiments 5 and 5b are especially compelling: counterintuitively, strong interference was restricted to the semantically *far* condition, whereas closely related distractors either generated a slight facilitatory effect (in Experiment 5), or an interference effect of less than half that of distantly related distractors (Experiment 5b). The explanation advocated by Mahon et al. is as follows: at the conceptual level, strongly related distractors cause larger facilitation than weakly related ones. At the “response buffer” level, only broad category membership is relevant, so strongly and weakly related distractors generate equivalent interference. The net outcome is that for strongly related distractors, conceptual priming, and response buffer interference largely cancel each other out; for weakly related distractors, relatively less conceptual priming results in a larger behavioral interference effect. PWI tasks should hence exhibit a “reversed semantic gradient.”

Overall, the role and impact of semantic overlap in PWI tasks remains somewhat inconclusive. The two studies by Vigliocco et al. (2004) and Mahon et al. (2007) rendered contradictory results which have never been satisfactorily accounted for. Other than these two key contributions, we are not aware of other studies which would have directly tested the effect of manipulating the semantic distance between categorically related pictures and words. Understanding the nature of within-category semantic gradients in the picture-word paradigm is an important issue which has critical implications for models of language production. A more complete understanding of the processes which contribute to naming response times is vital to progress debate as to whether lexical selection in word production is competitive or not.

In the work below, we contributed to the debate surrounding the nature of within-category semantic gradients in the PWI task by replicating the two key experiments by Vigliocco et al. (2004, Experiment 3) and Mahon et al. (2007, Experiments 5 and 5b). Both studies manipulated within-category distance in a similar manner, yet reported results which directly contradict each other. Hence, we deemed it important to re-run both experiments with the same apparatus and participant pool. Vigliocco et al. and Mahon et al. used the PWI task in a slightly differing format (outlined below); our aim was to run both studies with the same trial format hence our experiments are not exact replications of the original studies. The critical experimental aspects are as follows:

(1) Vigliocco et al. (2004) and Mahon et al. (2007) used picture-distractor SOAs of −150 and 0 ms, respectively. With visually presented distractors, an SOA of 0 ms is a popular and common choice when targeting semantic effects; in an analysis of the effects of varying SOAs in PWI tasks, Damian and Martin (1999) found the most pronounced semantic effect at this SOA, but reduced interference with −100 ms, and no interference with −200 ms. For this reason, we used SOA = 0 ms in both experiments reported below. (2) Both Vigliocco et al. and Mahon et al. presented target pictures centrally but varied distractor position slightly from trial to trial, although in different ways: Vigliocco et al. presented distractors in a randomly selected location either above or below the fixation cross (the degree of dislocation is not specified) whereas Mahon et al. varied distractor position both horizontally and vertically around the fixation cross by 2 cm. In our reading, the vast majority of published PWI studies with written distractors have used a central presentation of both targets and distractors, therefore we also used this format. We are not aware of findings in the literature suggesting that this procedural variation could be relevant, and indeed, the presence of semantic interference effects in both the previous studies and our own experiments clearly demonstrates that participants accessed the meaning of the distractors. (3) There was some minor variation in trial structure between the earlier studies: Vigliocco et al. presented a continuous series of trials to participants, with a trial sequence of: fixation cross for 500 ms, blank screen for 50 ms, distractor presented, target appearing 150 ms later, and both visible until response. In Mahon et al.'s studies, each trial was initiated by a participant via a key press; on each trial, a fixation cross was presented for 500 ms, followed by the target/distractor combination which was presented until the voice key detected a response. In our two experiments presented below, a continuous series of trials was delivered, and the trial sequence was as follows: a 1000 ms blank screen, followed by a centrally located fixation point presented for 500 ms, immediately followed in the same location by the target picture and distractor word for 2000 ms. There is no reason from the existing literature to suspect that such minor variations could affect results in PWI studies. (4) Neither Mahon et al. nor Vigliocco et al. stated the source of their target pictures for the critical experiments; however, Vigliocco et al. declared for an earlier experiment in their article that “pictures were obtained from Snodgrass and Vanderwart (1980) and supplemented by additional pictures created for the purpose” (p. 445). Indeed, 15/20 targets in Mahon et al., and 21/24 targets in Vigliocco et al. were in the Snodgrass and Vanderwart set. We therefore used these pictures, augmented with a few additional images selected from other object sets.

One aim of our experiments was to investigate whether we could replicate the central (and mutually contradictory) findings from the original studies. A second aim was to investigate the role of semantic overlap in PWI tasks at the item level, by computing interference scores for each target picture which identify the degree of semantic interference associated with a particular target-picture combination. We therefore explored the association between these item-specific interference scores and various measures of semantic relatedness, namely (1) semantic relatedness ratings which we collected from a separate group of participants, (2) semantic distance scores obtained via Latent Semantic Analysis (LSA), (3) Normalized Google Distance (NGD). By regressing item-specific interference effects onto these semantic relatedness measures, we expected to gain more detailed insight into the directionality (if any) of semantic effects in PWI tasks.

## Experiment 1

The first experiment aimed to provide a replication of Vigliocco et al.'s (2004) Experiment 3, with the procedural variation outlined above. Pictures were named in the presence of visually presented distractor words, with semantic distance between picture and word manipulated in four conditions: *far*, *medium*, *close*, and *very close*. The *far* condition corresponds to the unrelated condition used in numerous PWI studies. As Mahon et al. (2007; targeted in Experiment 2) used the label *far* to refer to a “weakly related” condition, from here on we will use the common label *unrelated* to avoid confusion. As discussed above, the relatedness between targets and distractors was established though FUSS (Vigliocco et al., 2004), with relatedness values for items in the *unrelated* group >18.5 units on the lexico-semantic map, in the *medium* group ranging from 7.5 to 10.5 units, the *close* group from 4.5 to 7.5 units, and the *very close* group from 1.5 to 4.5 units.

As pointed out by Mahon et al. (2007), some aspects of stimulus selection in this experiment are suboptimal (but justified on the basis of how Vigliocco et al., 2004, generated their materials). Although the same target pictures were used in each condition, the allocation of distractor words between the relatedness conditions was not as controlled, with many, but not all, words appearing in multiple distractor conditions. Further, although the bulk of distractors and pairs were categorically related, some of them were also associatively related (e.g., trousers-belt), some had form overlap (e.g., broom-banana), and indeed, a few pairs were not members of an obvious semantic category (e.g., axe-pencil). Nevertheless, the aim of the current research was primarily to establish the reliability of the original results, and consequently, no alterations were made to the original stimuli. To recap, the results of the original experiment identified a significant linear trend in which semantic interference increased with semantic overlap.

### Methods

#### Participants

Twenty-six undergraduate students at the University of Bristol were recruited as participants in the study and received course credit. For this and the following experiment, ethical approval was granted by the Faculty of Science Human Research Ethics Committee at the University of Bristol. All experiments conformed to the relevant regulatory standards. Informed consent was obtained from all participants prior to testing.

#### Materials

Materials were taken from Experiment 3 of Vigliocco et al. (2004), consisting of 24 target pictures paired with 67 distractor words to form *unrelated*, *medium*, *close*, and *very close* pairings. Note that as is typical of PWI studies, the same target pictures were used in all conditions, which excludes the possibility that between-item differences with respect to, e.g., the ability of object names to trigger the voice key, might obscure the results. The semantic distance between target and distractor was established by Vigliocco et al. through FUSS (described above) via semantic feature analysis. Target pictures were largely selected from a set previously shown to have high name agreement (Snodgrass and Vanderwart, 1980). Distractors were matched across the four conditions for frequency and length, and care was taken to minimize phonological overlap with targets, although, as noted above, this was not achieved across the entire stimuli set. Similarly to the original experiment, 24 filler pictures were selected from semantic categories other than those of the target set. Four distractor words were selected for each filler picture, one of which was selected on the basis of being semantically related to the filler picture while the other three were unrelated to the filler. The total number of experimental trials, including critical and filler items, was 192. Supplementary Material shows the critical combinations.

#### Design

Four experimental blocks were created from the 48 critical and filler pictures and the four distractors associated with each picture. In each block every picture was presented once. Across the four blocks, each picture was presented once with each of its four associated distractors and followed each other an equal number of times. Distractors were organized so that each experimental block contained a balanced number of each type, i.e., an equivalent proportion of *unrelated*, *very close*, *medium*, and *close* target-distractor pairs. The order of the blocks was manipulated so that each block was presented the same number of times in each position. Similarly to the original experiment, items were presented in a pseudorandom order with the only constraint stipulating that critical items and filler items alternated.

#### Procedure and apparatus

Participants were tested individually. Stimuli were presented using DMDX (Forster and Forster, 2003) running on a PC, with vocal responses captured by a head-mounted microphone. Prior to the commencement of testing participants were presented with two grid-like screens which contained all of the 48 targets pictures and their correct names. Participants were asked to familiarize themselves with the pictures until they felt they could name all of them with the given names (note that a pre-experimental familiarization phase, also used in Experiment 2, is common in PWI experiments, and was also used in the studies of Vigliocco et al., 2004; Mahon et al., 2007). A practice block followed in which each picture was presented once with an unrelated distractor word that was not used in the experiment proper. Participants were instructed to name the target pictures as quickly and accurately as possible, while ignoring written distractor words. In this phase, picture names other than those expected were corrected by the experimenter. Subsequently to the practice, the four blocks of experimental trials were presented. At the end of each block the experiment paused until the participant indicated they were ready to continue.

Targets and distractors were both presented centrally on the screen, and with the same onset (SOA = 0 ms). The same presentation and SOA were also used in our second experiment (see below), rendering them directly comparable. The sequence was as follows: a 1000 ms blank screen, followed by a centrally located fixation point presented for 500 ms, immediately followed in the same location by the target picture and distractor word for 2000 ms. Distractor words were presented in bold 18 pt Courier New typeface. All pictures were clearly visible despite the presence of the superimposed distractor word. DMDX recorded individual naming latencies to the harddrive and determined response latencies via a digital voice key relative to the onset of the target picture.

The entire session including the familiarization and practice process lasted approximately 30 min per participant.

### Results

#### Initial analysis

All responses were audiovisually checked for accuracy of the response trigger determined by DMDX, as well as for inaccurate responses using CheckVocal (Protopapas, 2007). Responses were classified as errors if, on a given trial, a name other than that of the target was produced, a correction was made, the response was disfluent, or no response was made within the response window. Latencies faster than 250 ms or longer than 1800 ms (3.6%) were excluded as outliers.

Table 1 shows the results, as well as the original Vigliocco et al. (2004) findings for comparison. For latencies, the results show a semantic interference effect of approximately 40 ms. Surprisingly this effect seems unaffected by the degree of semantic overlap between target and distractor, with very similar interference obtained for the *medium*, *close*, and *very close* conditions. Analyses of variance (ANOVAs) were conducted on the latencies, with either participants (*F*_1_) or items (*F*_2_) as the random variable, and Condition (*unrelated*, *medium*, *close*, *very close*) as a fixed variable. The results showed a highly significant effect of Condition, *F*_1(3, 75)_ = 11.65, MSE = 11,969, *p* < 0.001; *F*_2(3, 69)_ = 6.34, MSE = 10,093, *p* < 0.001. A trend analysis performed on the levels of Condition showed a combination of linear [*F*_1(1, 25)_ = 18.71, *p* < 0.001; *F*_2(1, 25)_ = 8.67, *p* = 0.007], quadratic [*F*_1(1, 25)_ = 8.94, *p* = 0.006; *F*_2(1, 25)_ = 12.07, *p* = 0.002] and cubic [*F*_1(1, 25)_ = 4.76, *p* = 0.039; *F*_2(1, 25)_ = 4.02, *p* = 0.056] components (by comparison, Vigliocco et al.'s results were characterized by an exclusively linear trend).

**Table 1:** **Mean response latencies (RT, in ms), error rates (PE, in %), and effects (related minus unrelated) for Experiment 1, and results from Vigliocco et al. (2004)**.

**Target-distractor relationship**	**RT**	**Effect**	**PE**	**Effect**
**EXPERIMENT 1**				
Unrelated	803 (101)		2.2 (3.7)	
Medium	846 (127)	43	2.7 (3.2)	0.5
Close	843 (130)	40	2.8 (5.4)	0.6
Very close	848 (110)	45	3.1 (5.5)	0.9
**Vigliocco et al., 2004, EXPERIMENT 3**				
Unrelated	642		5.9	
Medium	648	6	6.2	0.3
Close	657	15	6.1	0.2
Very close	671	29	7.5	1.6

Standard deviations are in parentheses.

Planned tests which compared the four conditions against each other showed that all three related conditions (*medium*, *close*, *very close*) differed significantly from the *unrelated* condition, *t*_1_ ≥ 4.63, *p* < 0.001; *t*_2_ ≥ 2.98, *p* ≤ 0.007, whereas the three related conditions did not differ significantly from each other, *t*_1_ ≥ 0.55, *p* ≤ 0.585; *t*_2_ ≥ 0.33, *p* ≤ 0.743. A further analysis was carried out subsequent to the removal of various potentially problematic stimuli (16 in total) that were either form related, associatively related, or not categorically related. Removal of these items failed to considerably affect results.

**Figure 1:**
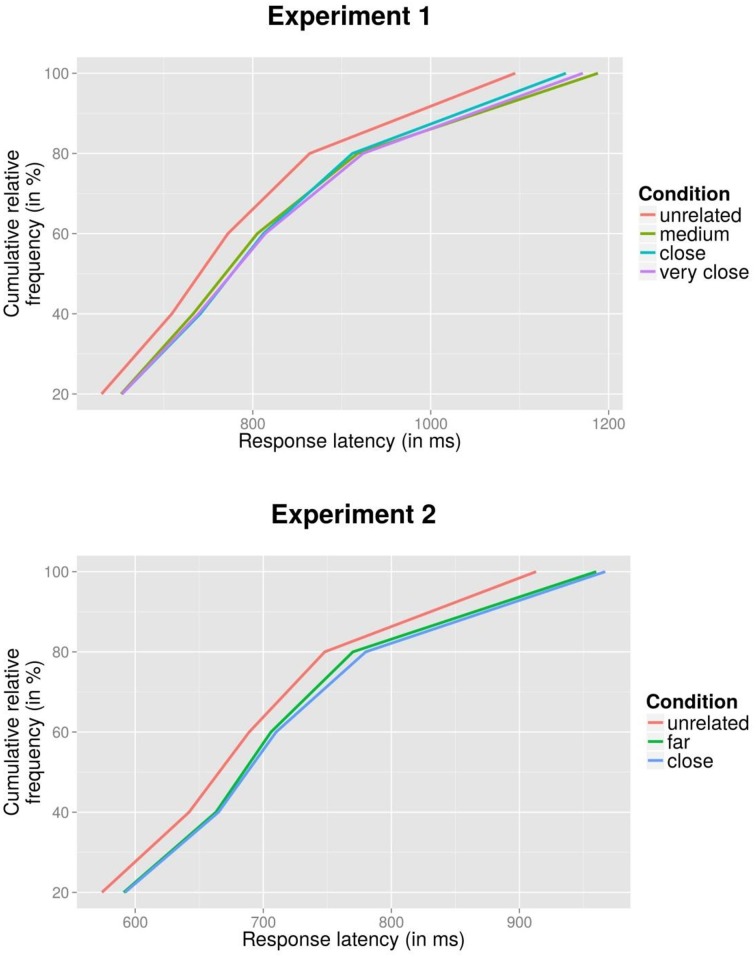
**Vincentized cumulative distribution curves for Experiment 1 (top panel) and Experiment 2 (bottom panel)**.

To further explore the effect of relatedness in the naming latencies, Vincentized cumulative distribution curves were computed (Ratcliff, 1979): for each participant and condition, rank-ordered latencies were divided into 20% quantiles, and mean latencies were computed for each quantile. These were then averaged across participants, which preserves the shapes of individuals' latency distributions (cf. Woodworth and Schlosberg, 1954). An analysis of this type provides information about the degree of uniformity with which an effect affects the spectrum of response latencies. This is particularly important in case of a null finding (here, no difference between the three related conditions): perhaps, very strongly related items show conceptual priming (which might manifest itself particularly in fast latencies) but also increased interference (which might particularly affect the right tail of latencies). In this case, the net result might not be visible in conditional means compared to less related conditions, but a pattern would emerge in response time distributions. Indeed, in the Stroop literature, null effects on mean latencies which result from different opposing underlying effects have been highlighted (e.g., Heathcote et al., 1991). Figure 1 (top panel) shows the results for the four conditions of Experiment 1: for all three related conditions, relatedness exerts a similar effect across the entire spectrum of response times compared to the baseline condition.

Parallel ANOVAs conducted on error proportions showed no effect of Condition, *F*_1_ < 1, *F*_2_ = 1.06.

In sum, the latency analysis showed a pattern in which the unrelated condition differed significantly from all three related conditions, but the extent of interference was not affected by the semantic distance between target and distractor. Next, we attempted to further elucidate the results by attempting to account for variability among individual targets-distractor pairs regarding their degree of semantic interference via a number of measures of semantic overlap. For each target and condition, we calculated the associated interference effect in the PWI study, and we assessed the fit between the interference effects and a range of measures of semantic overlap.

#### Semantic relatedness ratings

A straightforward way of identifying the degree of semantic overlap between a pair of items is to collect semantic relatedness ratings (e.g., Mahon et al., 2007). We conducted such ratings for all picture-distractor combinations in Experiment 1; items for Experiment 2 (described below) were also included. Twenty-seven individuals, none of whom were participants in the two experiments, were presented with pairs of words corresponding to pictures and distractors in the experiments, and were instructed to rate “how related the two concepts denoted by the words are” (these instructions were taken from Mahon et al.'s ratings). Ratings were carried out on a 1–7 scale, with seven indicating an “very related” pair and one a “not related” pair (a number of examples such as *spider-fly* and *house-bat* were provide as reference points for strongly related and unrelated pairs). A different random order of word pairs was presented to each participant. For the materials in the present study, mean relatedness ratings were 1.7, 4.5, 5.1, and 5.7 for the *unrelated*, *medium*, *close*, and *very close* conditions. From these, we calculated distance scores for each individual target picture by subtracting the unrelated baseline from each of the related ratings. For instance, the target picture “rake” has ratings of 1.9, 2.9, 3.6, and 5.6 when paired with the distractors “carpet,” “sword,” “hatchet,” and “shovel,” so relatedness scores are 1.0, 1.8, and 3.7 for the *medium*, *close*, and *very close* conditions. Hence, higher rating difference scores are associated with a stronger degree of relatedness (or more precisely, a larger difference between the related and the unrelated rating for that item).

Next, for each of the 24 target pictures, we calculated an “interference” score by subtracting the naming latency mean in the unrelated condition from each of the means in the related conditions. For instance, the target picture “rake” generates 51 ms interference when paired with the *medium* distractor “sword” (compared to when paired with the *unrelated* distractor “carpet”), 65 ms when paired with the *close* distractor “hatchet,” and 64 ms when paired with the *very close* distractor “shovel.” To account for variability between targets regarding their overall latencies, values were then converted into percentages relative to the *unrelated* baseline condition; e.g., for the target “rake,” *medium*, *close*, and *very close* conditions resulted in 5.4, 6.8, and 6.7% interference.

**Figure 2:**
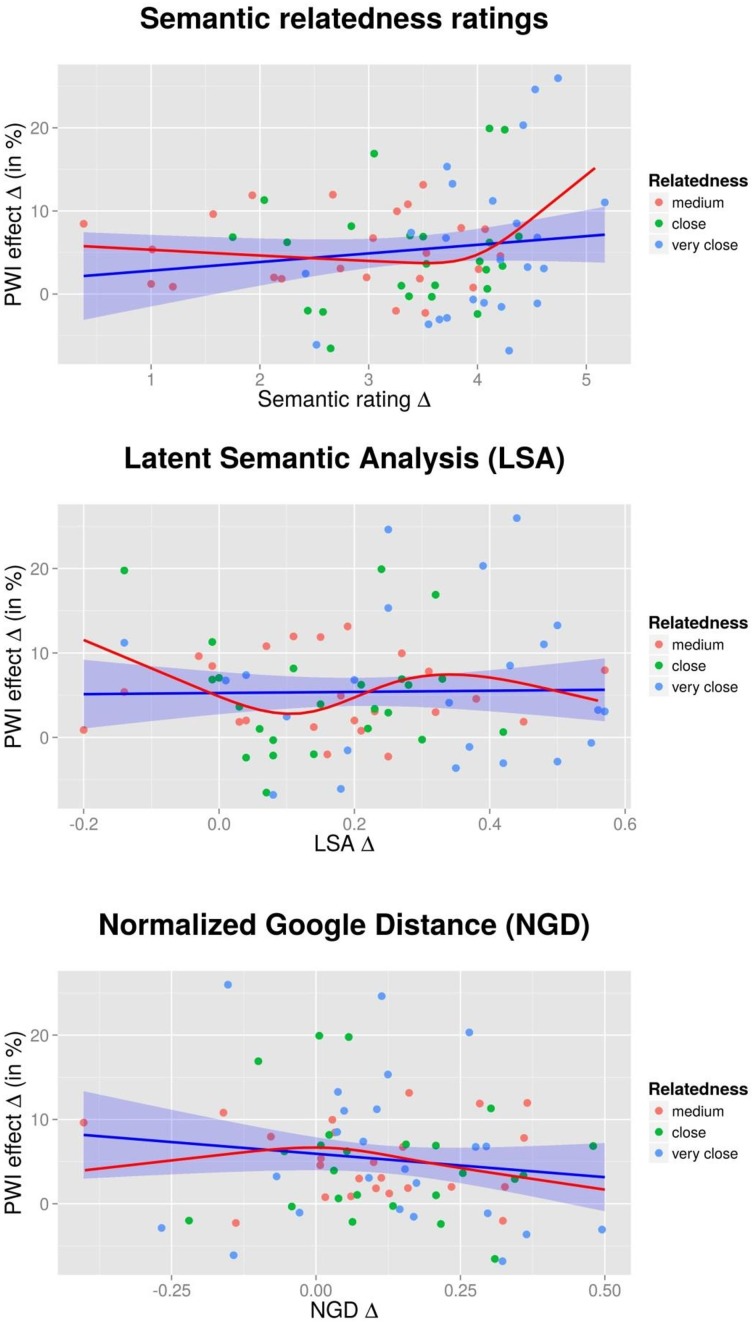
**Experiment 1—Degree of semantic interference (in percent), dependent on semantic relatedness ratings (top panel), Latent Semantic Analysis scores (LSA; middle panel), and Normalized Google Distance scores (NGD; bottom panel)**. Dots represent individual target-distractor combinations. The blue line corresponds to a regression between the two variables with linear terms; the red line corresponds to an analysis using restricted cubic splines (see text for details).

Figure 2 (upper panel) shows a scatter plots, with dots representing individual picture-word combinations (color-coded for condition). PWI interference is on the y-axis, and the ratings effect on the x-axis. If interference increases with growing semantic overlap (as predicted by Vigliocco et al., 2004) this should result in a positive slope; if interference is stronger for weakly related items (as stipulated by Mahon et al., 2007) a negative slope should emerge. A regression, with the trendline (plus confidence intervals) shown in blue was fitted to the data, but did not result in a significant outcome, *F*_(1, 70)_ = 1.55, *p* = 0.217, *R*^2^ = 0.022. However, inspection of a density plot of the residuals from the linear model suggested some degree of asymmetry, hence, we attempted to model this potential non-linearity via “restricted cubic splines” (RCS; Harrell, 2001; Baayen, 2008). This technique combines a series of cubic polynomials defined over a series of corresponding intervals. We chose four knots (i.e., three intervals) based on Harrell's suggestion for samples of our sizes (p. 135). Figure 2 (top panel) shows the outcome of the RCS analysis with a red line, suggesting a rise in the right tail end. A RCS model with 4 knots approached significance, *F*_(3, 68)_ = 2.49, *p* = 0.067, *R*^2^ = 0.10.

The RCS model suggests a possible tendency for a few very strongly related items (see upper right corner of the panel) to provide more interference (>20%) than the other, less related, items. The three combinations which generated interference larger than 20% are coat-suit, cucumber-broccoli, and finger-thumb. Note that the three target pictures *coat*, *cucumber*, and *finger* by themselves are not problematic, as they have average latencies in the unrelated condition which puts them below the overall unrelated mean (753, 788, and 693 ms).

We conclude that overall, semantic relatedness between picture and distractors—as assessed by semantic relatedness ratings—does not appear to affect the degree of semantic interference in the PWI task. The directionality of the trend emerging in the RCS analysis (increased interference for very strongly related items) is generally in line with the predictions made by Vigliocco et al. (2004), but the variance accounted for is low even with the RCS model (~10%).

#### Latent Semantic Analysis (LSA)

Vigliocco et al. (2004) selected their items via relatedness scores generated from FUSS, which are based on the number of shared semantic features. The analysis reported in the previous section showed no clear association between semantic relatedness ratings and the degree of interference in our PWI experiment. But perhaps ratings, based on individuals' intuitions about semantic relatedness, are not optimal to investigate conceptual structure, and “objective” measures do a better job in predicting PWI results. Although FUSS scores of the individual stimuli were not available to us, an alternative objective measure of semantic distance is Latent Semantic Analysis (LSA; Landauer et al., 1998). LSA applies statistical computations as a means of generating relatedness scores from a large corpus of text. The contextual usage of words is assessed through the aggregation of all the contexts in which a particular word does and does not appear, determining the similarity of meaning through a set of mutual constraints. The degree to which LSA reflects human knowledge has been demonstrated in a number of ways, including category judgment and word sorting (Landauer et al., 1998).

We computed LSA relatedness scores for each picture-target combination, in analog to what was described for the semantic relatedness ratings. As in Vigliocco et al. (2004, p. 448), we used the LSA web-based interface (http://lsa.colorado.edu/), using the “General reading up to 1st year of college” topic space and “Matrix comparison.” Then, LSA difference scores were computed in the same way as for the relatedness ratings described in the previous section, and plotted against behavioral interference effects (for three combinations, LSA scores were not available). Figure 2 (middle panel) shows the relationship between PWI interference and the difference in relatedness. A regression model representing a linear relation between LSA scores and interference did not result in a significant outcome, *F*_(1, 69)_ = 0.02, *p* = 0.884, *R*^2^ < 0.01, and neither did a RCS model with four knots, *F*_(3, 67)_ = 1.74, *p* = 0.167, *R*^2^ = 0.07.

#### Normalized Google Distance (NGD)

We attempted to predict the amount of interference via “Normalized Google Distance” (NGD), a semantic similarity measure derived from the number of hits returned by the Google search engine for a given set of words (Cilibrasi and Vitanyi, 2006, 2007). The normalized Google distance between two search terms *x* and *y* is computed as

$$\text{NGD}(x,y)=\frac{\text{max}\{\log f\left(x\right),\log f\;(y)\}-\text{log }f(x,y)}{\log M-\text{min}\{\log f\left(x\right),\log f(y)\}}$$

with *M* the total number of pages available to Google, *f*(*x*) and *f*(*y*) the number of hits for individual search terms *x* and *y*, and *f*(*x, y*) the number of hits for joint occurrence. Words which tend to co-occur in the search space take on values close to zero, whereas words which never co-occur take on infinite values: words with similar meaning tend to be close (have lower values) than words with dissimilar meaning. For instance, “coat” and “suit” tend to co-occur (NGD = 0.01) whereas “coat” and “bus” do so less often (NGD = 0.26).

We computed NGD values for all picture-distractor combinations used in Experiment 1^1^. All three related conditions resulted in quite similar average NGD values (0.22, 0.19, and 0.19 for the *medium*, *close*, and *very close* condition) which were slightly lower than those for the unrelated condition (0.31). As for relatedness ratings and LSA measures, we then computed difference scores for all related, relative to the unrelated, combinations. Here we subtracted the related from the unrelated condition (rather than vice versa, as in the previous two analyses) to preserve the directionality of the other two analysis, i.e., higher NGD difference values reflect stronger overlap. Figure 2 (bottom panel) shows the relationship between PWI interference and the NGD difference scores. A regression model with a linear relation between NGD and interference did not show a significant outcome, *F*_(1, 70)_ = 1.34, *p* = 0.251, *R*^2^ = 0.02, and neither did a RCS model with four knots, *F*_(3, 68)_ = 0.83, *p* = 0.481, *R*^2^ = 0.04.

### Discussion

To summarize the results, a clear and strong effect of semantic relatedness was found in this experiment, in line with numerous published studies in the literature. Surprisingly, however, when the unrelated group was compared to the three related conditions, there was no evidence to suggest that response latencies varied as a function of semantic distance. Quantile plots demonstrated that in each of the related conditions, latencies increased uniformly across the entire range of responses relative to the baseline condition, with very little or no difference between them. Consequently, our findings did not suggest the presence of a semantic gradient in which more closely related targets and distractors result in greater interference.

We collected semantic relatedness ratings on our items, and tried to predict the size of the interference effect for a particular target-distractor combination, depending on their rated relatedness. A marginally significant pattern was found when ratings were modeled onto interference effects via a non-linear technique, with pronounced interference for very strongly related items. More remarkable, however, is the null finding for all but those few items. With two further, alternative measures of semantic distance, namely LSA- and NGD-derived scores of overlap, no systematic pattern was found. Overall, the results are remarkable in their absence of an effect of degree of semantic overlap, despite the presence of strong and significant effects of relatedness when compared to the unrelated baseline.

## Experiment 2

Experiment 1 provided no compelling evidence for a semantic gradient in PWI tasks. This contrasts with Vigliocco et al. (2004) original experiment where a statistically significant linear trend indicated that more closely related distractors slowed naming more than distantly related items. The second experiment constituted an attempt to replicate (again, with minor procedural variations as described in the Introduction) perhaps the most compelling evidence for a “reversed semantic gradient,” i.e., distantly related distractors interfere *more* with picture naming than closely related distractors. As described in the Introduction, Mahon et al. (2007, Experiments 5 and 5b) compared an *unrelated* baseline to a condition in which items were distantly related (*far*) as well as one in which they were closely related (*close*), and found significant interference only in the *far* condition, but no (Experiment 5) or substantially reduced (Experiment 5b) interference for the *close* condition. In each condition, the same target pictures were named, and the same distractor words used (but differently combined with the targets). Strength of relatedness was established via semantic relatedness ratings. Our Experiment 2 replicates this study, with the only modification other than those outlined in the Introduction the exclusion of a small number of items, for reasons outlined in the Section “Materials.”

### Methods

#### Participants

Sixty-four undergraduate students at the University of Bristol were recruited as participants and received course credit. None had been in the first experiment.

#### Materials

The stimuli were taken from Mahon et al.'s (2007) Experiments 5 and 5b. The majority of target pictures were from the Snodgrass and Vanderwart (1980) set. Of the original 20 target pictures, two (*boat* and *plane*) were removed because the corresponding related distractor words *submarine* and *helicopter* were relatively long (materials were originally selected with the intention to be included in a study using masked priming, in which long distractor words would be problematic). A further target, *plate*, had a high error rate in a pilot study because it was highly confusable with its corresponding distractor *saucer*, and was therefore omitted. Due to the way in which materials were arranged (see below) this required the removal of an additional target, *glass*.

The remaining 16 target pictures were paired with 16 categorically related distractor words. This set of distractors was recombined with targets to manipulate semantic distance. As in Mahon et al. (2007), target pictures were chosen in pairs from a particular semantic category (e.g., furniture: *bed* and *stool*), each paired with a closely related distractor word (*close*: bed-futon; stool-chair), and the distractors reversed to form more distantly related combinations (*far*: bed-chair; stool-futon), and finally paired with unrelated distractors (*unrelated*: bed-pot; stool-zebra) which themselves served as related distractors when combined with other targets. This arrangement allowed the use of the same set of 16 distractor words across all conditions. Note that this design necessitated two unrelated conditions. In total there were 64 target-distractor pairings. See Supplementary Material for all critical combinations.

For targets and distractors in the *close* and *far* conditions, the original arrangement from Mahon et al. (2007) was maintained. As some targets and distractors had been removed from the original stimulus set, a number of the original unrelated pairings were no longer possible, and we recombined items for the unrelated condition. Care was taken to ensure that pairs in this condition were associatively, categorically, and phonologically unrelated.

#### Design

The 64 trials were split into two blocks, such that each half contained an equal number of *unrelated*, *close*, and *far* items. There were two instances of each distractor and each target picture in each half of the stimuli. The order in which participants were presented with each half of the stimuli was counterbalanced. Presentation order was randomized, with a minimum distance of three trials between the first and second presentation of each target picture and distractor within each block, as well as a maximum of three consecutive related or unrelated target-distractor pairs.

#### Procedure and apparatus

The procedure was very similar to Experiment 1. Prior to the experiment, participants were familiarized with the critical target pictures with via a grid-like screen with the 16 target pictures and their names. A practice block followed in which each picture was presented once with an unrelated distractor word that was not used in the experiment proper; responses other than those expected were corrected by the experimenter. Subsequently, the two experimental blocks, each consisting of 32 trials, were presented. At the end of each block the experiment would pause until the participant was ready for the next block.

The same presentation sequence as in Experiment 1 was used (1000 ms blank screen; 500 ms fixation cross; target and picture simultaneously presented for 2000 ms). Distractor words were presented in bold 18 pt Courier New typeface.

The entire session including the familiarization and practice phase lasted approximately 15 min per participant.

### Results

#### Initial analysis

Data were processed in the same way as in the first experiment. Latencies faster than 250 ms or longer than 1800 ms (2.1%) were excluded as outliers. Table 2 shows the results, together with the corresponding results from Mahon et al. (2007). Latencies showed approximately 20 ms of semantic interference, and very similar degrees of semantic interference for the *far* and *close* conditions relative to the *unrelated* baseline (for this and all following analyses, the two unrelated baselines were averaged).

**Table 2:** **Mean response latencies (RT, in ms), error rates (PE, in %), and effects (related minus unrelated) for Experiment 2, and results from Mahon et al. (2007)**.

**Target-distractor relationship**	**RT**	**Effect**	**PE**	**Effect**
**EXPERIMENT 2**				
Unrelated	708 (94)		1.5 (3.0)	
Far	727 (112)	19	2.2 (3.6)	0.7
Close	731 (109)	23	2.4 (4.2)	1.4
**Mahon etal., 2007, EXPERIMENT 5**				
Unrelated	728		1.2	
Far	765	37	1.8	0.6
Close	724	-4	1.9	0.7
**Mahon etal., 2007, EXPERIMENT 5b**				
Unrelated	709		1.6	
Far	746	37	2.0	0.4
Close	726	17	2.4	0.8

ANOVAs applied to latencies, with Condition (*unrelated*, *far*, *close*) as a fixed variable, showed a highly significant effect of Condition by participants, *F*_1(2, 126)_ = 9.07, MSE = 12028, *p* < 0.001, which was marginally significant in the analysis by items, *F*_2(2, 30)_ = 2.85, MSE = 2456, *p* = 0.074. We did not perform a trend analysis as in the first experiment, due to the low number of conditional means. Planned tests which compared the two conditions against each other showed that the two related conditions (*far*, *close*) differed significantly from the unrelated condition in the analysis by participants, *t*_1_ ≥ 3.35, *p* ≤ 0.001, and marginally in the analysis by items, *t*_2_ ≥ 1.78, *p* ≤ 0.095. By contrast, the two related conditions did not differ significantly from each other, *t*_1_ = 0.67, *p* = 0.503; *t*_2_ = 0.48, *p* = 0.636. Figure 1 (bottom panel) shows cumulative response time distributions which suggest a similar effect for the two related conditions compared to the unrelated condition across the entire spectrum of response times.

Parallel ANOVAs conducted on error proportions showed an effect of Condition which was significant by participants, *F*_1(2, 126)_ = 3.12, MSE = 34.3, *p* = 0.048, but not by items, *F*_2(2, 30)_ = 2.32, MSE = 8.6, *p* = 0.116. Planned tests showed that the *close* condition differed significantly from the *unrelated* condition in the analysis by participants, *t*_1(63)_ = 2.16, *p* = 0.034, and just failed to reach significance by items, *t*_2(15)_ = 2.12, *p* = 0.051. The *far* condition also differed significantly from the *unrelated* condition in the analysis by participants, *t*_1(63)_ = 2.05, *p* = 0.045, but not by items, *t*_2(15)_ = 1.46, *p* = 0.164. The two related conditions did not differ significantly from each other, *t*_1(63)_ = 1.04, *p* = 0.300; *t*_2(15)_ = 0.86, *p* = 0.403.

#### Semantic relatedness ratings

The materials used in this experiment had been included in the semantic relatedness ratings outlined in Section Semantic relatedness ratings. The results showed average ratings of 1.6 for the *unrelated* condition, of 4.9 for the *far* condition, and of 6.1 for the *close* condition. This compares well with the relatedness rating results reported in Mahon et al. (2007), which had shown means of 1.3, 3.9, and 5.3.

**Figure 3:**
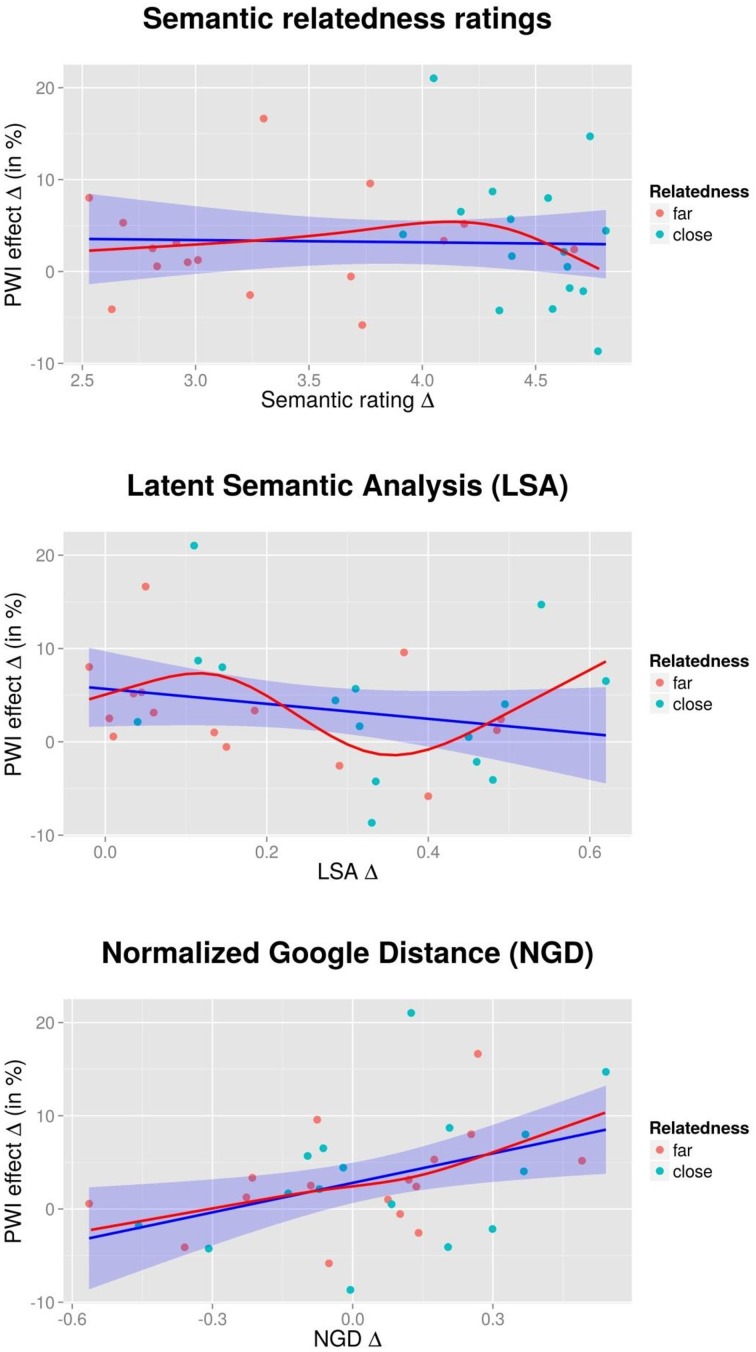
**Experiment 2—Degree of semantic interference (in percent), dependent on semantic relatedness ratings (top panel), Latent Semantic Analysis scores (LSA; middle panel), and Normalized Google Distance scores (NGD; bottom panel)**. Dots represent individual target-distractor combinations. The blue line corresponds to a regression between the two variables with linear terms; the red line corresponds to an analysis using restricted cubic splines (see text for details).

As in the first experiments, the association between ratings and corresponding PWI effects were investigated. Rating and PWI effects for each picture-word combination were computed in the same way as described in Section Semantic relatedness ratings. Figure 3 (top panel) shows the results. A regression model with a linear relation between ratings and PWI effects resulted in no significant outcome, *F*_(1, 30)_ = 0.03, *p* = 0.873, *R*^2^ < 0.01, and neither did a RCS model with four knots, *F*_(3, 28)_ = 0.59, *p* = 0.627, *R*^2^ = 0.06.

#### Latent Semantic Analysis (LSA)

LSA scores were computed for each target-distractor combination, and difference scores were calculated in the same way as outlined in Section Latent Semantic Analysis (LSA). Figure 3 (middle panel) shows the association between LSA difference scores and behavioral PWI effects. A regression model with a linear relation resulted in no significant outcome, *F*_(1, 28)_ = 1.69, *p* = 0.204, *R*^2^ = 0.06. A RCS model with four knots resulted in a marginally significant outcome, *F*_(3, 26)_ = 2.92, *p* = 0.053, *R*^2^ = 0.25.

#### Normalized Google Distance (NGD)

We computed NGD scores for the current stimuli, as described under Section Normalized Google Distance (NGD). Average values were 0.35 for the *unrelated* condition, and 0.33 and 0.28 for *far* and *close* combinations, respectively. Figure 3 (bottom panel) shows the relationship between NGD difference scores and behavioral PWI effects. A regression model with a linear relation resulted in a significant outcome, *F*_(1, 30)_ = 6.61, *p* = 0.015, *R*^2^ = 0.18. By contrast, a RCS model with four knots was not significant, *F*_(3, 28)_ = 2.20, *p* = 0.111, *R*^2^ = 0.19.

### Discussion

In this experiment, an effect of relatedness emerged both in the latency and error analyses: in line with numerous published results in the literature, categorically related distractors interfered with target naming. As in the first experiment, however, no clear pattern emerged with regard to a semantic gradient: *close* and *far* conditions had quite similar average latencies, suggesting that the degree of overlap was largely irrelevant. Cumulative response time plots confirmed this pattern, with both related conditions slowing down responses across the entire spectrum. Furthermore, we explored whether the size of the interference effect generated by a particular picture-distractor combination could be predicted based on various measures of semantic relatedness. Relatedness ratings had no predictive power. LSA scores did not predict interference in a linear analysis, but showed a marginally significant result when modeled via RCS. NGD scores significantly predicted interference in the linear analysis. Overall, the pattern is that as in the first experiment, remarkably little variability in the PWI task is explained by measures of semantic overlap between target and distractor.

## General discussion

In two experiments we sought to replicate mutually contradictory previous findings regarding the possibility of a “semantic gradient” in PWI tasks: do strongly related targets and distractors induce more semantic interference than weakly related ones, as previously reported by Vigliocco et al. (2004), or does the opposite hold, as reported by Mahon et al. (2007)? Answering this question is of critical importance for the recent debate on whether lexical selection in spoken production is competitive or not. We duplicated the two previous key empirical studies with only very minor modifications (see Introduction) and found a general semantic interference effect: in both experiments, semantically related distractors slowed picture naming times, relative to unrelated distractors. However, in neither study did we find an additional semantic gradient; the size of the semantic interference was not influenced by whether items were strongly or weakly related.

Our attempts to model a linear or non-linear relationship between interference effects and various measures of semantic relatedness rendered mixed results. In Experiment 1, only the non-linear relationship between semantic relatedness measures and interference approached statistical significance. Figure 2 (top panel; red line) suggests that the degree of relatedness is largely irrelevant for the measured extent of PWI interference, except for a few items which are particularly strongly related and entail large interference effects. However, the corresponding analysis on the items from Experiment 2 show a different outcome, with (if at all) strongly related items creating *less* interference (although the RCS analysis was very far from significance). Could it be that the most strongly related target-distractor pairs in Experiment 2 were more related than the most strongly related pairs in Experiment 1? The relatedness ratings that we collected do not suggest that this was the case: average ratings were 5.7/7 for the *very close* condition in Experiment 1, and 6.1/7 for the *close* condition in Experiment 2. Direct comparison of the topmost panels of Figures 2 and 3 also shows that strength of semantic overlap for the most strongly related pairs is quite similar.

In Experiment 2, two further results from the regression analysis were significant (or close to significance): first, an RCS model with LSA scores as the predictor rendered a marginally significant result. However, the pattern (red line in the middle panel of Figure 3) is not easily interpretable, and in any case does not resemble the likewise curve from Experiment 1 (red line in the middle panel of Figure 2). Second, a regression with a linear relation between NGD and interference returned a significant result (bottom panel of Figure 3). This pattern (more interference for items with stronger overlap as measured by NGD) goes against the predictions from the REH, but a similar pattern was not found in the first experiment (bottom panel of Figure 2). Overall, the most striking aspect of the regression results is how little of the variance is accounted for by any of the semantic relatedness measures, with all *R*^2^ ≤ 0.25.

These results admittedly are somewhat perplexing. Not only did the two existing key studies by Vigliocco and Mahon report contradictory results, but our own attempts to replicate them rendered results which are not compatible with either of the earlier findings (but results were consistent across our two experiments, such that in both experiments semantic interference was found, coupled with little additional effect of semantic relatedness). For the time being tentatively accepting our null finding concerning the effects of semantic distance in PWI tasks, how can the results be interpreted, and what are the implications for the current debate on whether or not lexical selection in spoken production is competitive?

A central component of REH is the claim that the response buffer is sensitive only to categorical membership but not to the degree of semantic similarity. Hence at this level both weakly and strongly related picture-word distractors should generate identical relatedness effects, as was found in Experiment 2 where close and far conditions had very similar latency means. Additionally, REH assumes that for strongly related items, conceptual priming counteracts response buffer-based interference^2^. Given our null finding concerning a semantic gradient, a possibility for advocates of REH would be to drop the claim that there is conceptual priming, and state that semantic effects in PWI are exclusively response buffer-based. This is possible, but would then leave unexplained why some forms of semantic overlap (associatively related, part-whole, etc.; see Introduction) tend to generate facilitation effects in PWI tasks, given that conceptual priming was hypothesized to be the source of such effects.

Similarly, however, the results are not straightforwardly explained within the typical assumptions of competitive lexical selection. Although we are not aware of attempts to simulate the effects of varying semantic overlap in models of this type (such as WEAVER++), intuitively strongly related distractors should cause more interference than weakly related ones. Given the possibility that effects in PWI could reflect a combination of conceptual priming and lexical competition, perhaps models of this type could be specified such that the two contradictory forces cancel each other out: strongly related distractors cause substantial conceptual priming which facilitates target retrieval, yet also induce powerful competition which slows down target retrieval; weakly related distractors cause relatively less conceptual priming, but also less lexical competition. Only detailed computational simulations will show whether this possibility is feasible.

Finally, our results do not provide clear support for an alternative account of distractor interference which predicts greater interference from distantly related category members while maintaining the assumption of competitive lexical selection (Abdel Rahman and Melinger, 2009). Following this account, more closely related distractors should lead to a smaller cohort of items becoming activated during lexical selection, and consequently, less interference should be observed in comparison to the larger cohorts activation when more distantly related category members are presented. However, our results do not show the predicted negative semantic gradient.

As outlined in the Introduction, when setting up our studies we modified a number of relatively minor procedural details compared to the original experiments, mainly in order to render the two studies more similar and therefore comparable to each other. Based on the extensive literature on the PWI technique, there is no reason to believe that these variations could have critically influenced the results. For instance, Vigliocco et al. (2004) used an SOA of −150 ms, whereas we used 0 ms. Could it be that under the negative SOA, a semantic gradient is present (as suggested by Vigliocco et al.), whereas under a simultaneous SOA degree of relatedness is not relevant (as suggested by our results)? Although this is not impossible, it would be a challenge to account for such a pattern within the theoretical frameworks currently available to explain PWI effects. Nevertheless, future work should perhaps aim to replicate the original studies to a closer extent than we accomplished.

A central component of our research approach was the attempt to predict semantic effects for individual picture-word pairs, based on a range of measures of semantic overlap. Human semantic relatedness ratings showed a reasonable degree of association with values obtained from LSA (Experiment 1: *r* = 0.564, *p* < 0.001; Experiment 2: *r* = 0.521, *p* = 0.003). However, one aspect of the findings which came as a surprise is that there was particularly low convergence between Normalized Google Distance (NGD) and the other two measures: NGD did not correlate with ratings (Experiment 1: *r* = 0.035, *p* = 0.772; Experiment 2: *r* = 0.183, *p* = 0.316) nor with LSA scores (Experiment 1: *r* = −0.098, *p* = 0.415; Experiment 2: *r* = −0.044, *p* = 0.819). Given the claim that NGD allows the automated discovery of meaning (Cilibrasi and Vitanyi, 2006, 2007), it is surprising that these correlations are so low. We conclude that NGD evidently gauges a different construct from the other two types of measures, one which is primarily sensitive to co-occurrence rather than to overlap in terms of semantic properties (indeed, the three related conditions in our first experiment had virtually identical NGD scores; the two related conditions in the second experiment were also relatively similar to each other).

A few other aspects of the results deserve discussion. In our second experiment, overall latencies (708 ms in the unrelated condition) were very similar to those reported by Mahon et al. (2007; 719 ms across their Experiments 5 and 5b). By contrast, there is a relatively large discrepancy between the unrelated mean of our first experiment (802 ms) and the one reported by Vigliocco et al. (642 ms). One possible contributing factor is that Vigliocco et al. used an SOA of −150 ms, whereas we used one of 0 ms. It is well known in PWI studies that the mere presence of a visually presented distractor word (irrespective of semantic or form overlap with the target) tends to maximally interfere with target naming when onset of both target and distractor coincide. For instance, Damian and Martin (1999, Experiment 1) reported unrelated means of 700, 714, and 744 ms for SOAs of −200, −100, and 0 ms. This gradient is most likely attributable to attentional interference when two stimulus dimensions are presented in extremely close succession or simultaneously.

A further aspect worth noting is that we observed a considerable difference in the latencies between our two experiments (an unrelated mean of 708 ms in Experiment 1, and one of 802 ms in Experiment 2). Given that we obtained target pictures mostly from the Snodgrass and Vanderwart (1980) set, recruited participants from the same pool, and aimed to render the two experiments procedurally as similar as possible, the reason for this speed difference is currently unclear. Finally, it must be noted that the size of the semantic effect in Experiment 1 (ignoring the degree of relatedness) was 40 ms, but in Experiment 2 it was only 21 ms. Again, the reasons for this variation are unclear. It is not implausible that overall slower latencies (as in the first, compared to the second, experiment) should be associated with larger semantic interference effects, but this is unlikely to account for the observed size difference.

The lack of an effect of strength of relatedness in both experiments could be considered a null finding: the amount of interference generated by semantically related distractors in PWI is not influenced by semantic relatedness. Given the general difficulty in interpreting null findings, we sought to further explore the results of Experiment 1 via calculation of Bayes factors (Dienes, 2011), with the following line of reasoning. In Vigliocco et al., compared to the full-blown interference effect in the “very close” condition (29 ms), effects in the “close” and “medium” condition (15 and 6 ms, respectively) were reduced by 14 and 23 ms, or by 48 and 79% of the maximal value in the “very close” condition. In our own results, the “very close” condition resulted in interference of 42 ms; a reduction of this effect for the “close” and “medium” condition of the same size as found in Vigliocco et al. would predict values of 22 and 33 ms. Given the empirically obtained effects (reduction of 5 ms for the “close,” and of 0 ms for the “medium” condition) and corresponding standard errors, we were able to calculate Bayes Factors for the two conditions. We used the effects predicted from Vigliocco et al. as the mean of a normal distribution, with a standard deviation half that size as suggested by Dienes (2011, Supplementary Material). This resulted in Bayes factors of 0.77 for the “close” condition, and 0.37 for the “medium” condition. Based on the convention that Bayes factors at or below 1/3 are considered as substantial evidence supporting the null hypothesis, we argue that this is definitely the case for the “medium” condition, and less conclusively so for the “close” condition. A similar analysis was conducted on the results of Experiment 2: a Bayes Factor was calculated for the null difference between the effects generated by the “far” and the “close” condition. In Mahon et al. (averaged across their Experiments 5 and 5b), the effect for the “close” (7 ms) condition was reduced by 30 ms, or 82%, relative to the “far” condition (37 ms). For our own results this would predict an effect of 3 ms for the “close” condition (we found 23 ms). Given the same assumptions as in the corresponding analysis in Experiment 1, we obtained a Bayes Factor of 0.13, which constitutes strong evidence supporting the null hypothesis.

Additionally, critics might argue that the evidence provided here is inconclusive due to potential power issues. For the omnibus ANOVAs, the calculated *post-hoc* power to detect a medium-sized (0.25) effect given the included number of participants and conditions was 0.54 in Experiment 1, and 0.88 in Experiment 2. The regression analyses described in Sections Semantic relatedness ratings, Latent Semantic Analysis (LSA), and Normalized Google Distance (NGD) corresponded to analyses by items, hence, power is determined by the number of combinations which were included in the design, rather than the number of participants tested. For Experiment 1, an analysis returned a power value of 0.73 to determine a medium-sized (0.3) effect with this sample size. In Experiment 2, fewer items were included, and the power was 0.38, which is admittedly low. Note that as these two experiments sought to replicate the earlier studies by Vigliocco et al. (2004) and Mahon et al. (2007), we were restricted to using the original materials. Future work on the potential role of semantic distance effects in PWI tasks would be well advised to substantially enlarge the included number of items (in addition to testing a large number of participants) in order to minimize the chances of type II errors.

Given that we were not able to fully resolve the issue of the role of semantic overlap in PWI tasks, the question arises of what could be the next step in tackling the problem. On balance, the design of Experiment 2 in which the same distractor words are used across all conditions is clearly preferable to the one in Experiment 1 in which only some, but not all, distractor words re-occurred across conditions. However, the number of items which we included in our Experiment 2 (see Section Materials) was admittedly low, and it would be desirable to replicate the design of this study with a considerably increased number of items. We believe that researchers should also consider an approach involving multiple regression, in which a large number of targets and distractors are shown in various combinations, category membership is binarily coded, and the question is to what extent residual variance in latencies can be attributed to semantic overlap measures derived from ratings etc. once category membership is taken into account. Overall, the issue of whether degree of semantic overlap matters in PWI tasks remains remarkably elusive, and we do not believe that the currently available results should be used to constrain theorizing on the nature of lexical selection in spoken word production.

### Conflict of interest statement

The authors declare that the research was conducted in the absence of any commercial or financial relationships that could be construed as a potential conflict of interest.

## Footnotes

^1^ Normalized Google distance (NGD) values were computed in March 2014, and are based on http://www.google.com

^2^ On a strict reading of the response exclusion hypothesis, the assumption that target naming is delayed until the distractor has been removed from the response buffer predicts that latencies should exclusively depend on distractor processing, and any effects associated with target processing should be obliterated (Mulatti and Coltheart, 2012). For instance, the well-documented frequency effect in object naming (e.g., Jescheniak and Levelt, 1994) should disappear in a PWI context because processing of both high- and low-frequency target names are delayed until the distractor has been purged; however, that is clearly not the case (e.g., Miozzo and Caramazza, 2003, Experiment 1). Likewise, if target processing is speeded up due to conceptual priming, this should not affect latencies in a PWI task because the target will still have to wait until the distractor has been purged from the buffer.
