# Supplementary material for: Semantic gradients in picture-word interference tasks: is the size of interference effects affected by the degree of semantic overlap?
Source: Front Psychol. 2014 Aug 12;5:872. doi: 10.3389/fpsyg.2014.00872 (PMC4130197; doi:10.3389/fpsyg.2014.00872)
Supplement: Supplementary file 1 [file DataSheet1.DOCX]

*Appendix A*

Materials used in Experiment 1

| Target | Distractor | | | |
| --- | --- | --- | --- | --- |
|  | unrelated | medium | close | very close |
| apple | raft | bean | lemon | peach |
| axe | ceiling | pencil | spanner | hammer |
| banana | broom | potato | melon | peach |
| camel | sofa | swan | mouse | zebra |
| celery | dustpan | watermelon | mushroom | aubergine |
| cherry | spoon | bean | lemon | pear |
| coat | bus | belt | shoe | suit |
| corn | sofa | pear | pea | bean |
| cucumber | shield | strawberry | pumpkin | broccoli |
| dog | comb | duck | tiger | rabbit |
| fence | bus | roof | wall | gate |
| finger | couch | knee | wrist | thumb |
| hand | bus | thumb | leg | arm |
| hat | bomb | belt | shoe | scarf |
| hoe | tricycle | tweezers | hatchet | chisel |
| lettuce | rug | lime | pepper | onion |
| orange | dustpan | pumpkin | raisin | plum |
| pig | bomb | duck | lion | goat |
| pliers | tricycle | scissors | hatchet | hammer |
| rake | carpet | sword | hatchet | shovel |
| saw | curtain | pencil | drill | hammer |
| screwdriver | feather | sword | hatchet | chisel |
| sheep | beak | swan | zebra | goat |
| trousers | couch | belt | glove | shirt |

*Appendix B*

Materials used in Experiment 2

| Target | Distractor | | | |
| --- | --- | --- | --- | --- |
|  | close | far | unrelated A | unrelated B |
| bed | futon | chair | pot | zebra |
| stool | chair | futon | zebra | pot |
| dog | wolf | lizard | grenade | mallet |
| snake | lizard | wolf | mallet | grenade |
| horse | zebra | whale | chair | spoon |
| dolphin | whale | zebra | spoon | chair |
| pliers | wrench | mallet | shrub | lizard |
| hammer | mallet | wrench | lizard | shrub |
| kettle | pot | spoon | futon | whale |
| ladle | spoon | pot | whale | futon |
| tree | shrub | grass | wrench | wagon |
| flower | grass | shrub | wagon | wrench |
| car | truck | wagon | spear | grass |
| carriage | wagon | truck | grass | spear |
| bomb | grenade | spear | wolf | truck |
| arrow | spear | grenade | truck | wolf |
